# Intraoperative Diagnosis of Gallbladder Volvulus

**DOI:** 10.1155/2023/1194077

**Published:** 2023-10-31

**Authors:** Hassan Mohamed Ali, Eyad Rabih Abdul Wahab, Amjad Damaj, Bassam Moussawi, Walid Audi, Mohammad Haidar, Youssef Badra

**Affiliations:** ^1^Universite Saint-Joseph-Beirut, Beirut, Lebanon; ^2^Aston University College of Health and Life Sciences, Birmingham, UK; ^3^Lebanese University, Beirut, Lebanon; ^4^Dar Al Amal University Hospital, Douris, Lebanon; ^5^American University of Beirut Medical Center, Beirut, Lebanon

## Abstract

**Background:**

A gallbladder torsion typically presents as an acute abdomen presentation with nonspecific clinical signs. When not detected by a clinician preoperatively, it can lead to the delay of emergency surgery and possible misdiagnosis for other, more common causes of an acute abdomen. *Case Presentation*. We report the case of acute gallbladder volvulus in an 80-year-old woman complaining of constant pain in the right upper quadrant of the abdomen.

**Conclusions:**

The patient was successfully treated with cholecystectomy. The case highlights the high index of suspicion required to diagnose the condition preoperatively in this population of patients to reduce complications such as biliary peritonitis and death.

## 1. Background

The gallbladder volvulus is defined as the rotation of the gallbladder on its mesentery along the axis of the cystic duct and cystic artery [1]. Since its first description in 1898 by Wendel, only 500 cases were reported. Gallbladder torsion frequently occurs in the elderly, with the highest occurrence in the 65-75-year-old age group and a higher predilection (3 : 1) for women than men. Its rare presentation and frequent misdiagnosis have given it the name “acute cholecystitis with a twist” [2]. Unsurprisingly, it is reported that very few cases are diagnosed preoperatively, thus management is delayed. This life-threatening surgical emergency is frequently misdiagnosed as acute cholecystitis due to its comparable clinical picture, and this leads to clinicians delaying the critical cholecystectomy. This delay subsequently increases the risk of sepsis secondary to gallbladder ischemia, necrosis, and perforation [3]. Imaging studies have been described as providing nonspecific findings which again makes early detection difficult. Ultrasound imaging typically shows a distended gallbladder inferior to its normal anatomical position with a thickened wall and surrounded by free fluid [4]. Magnetic resonance (MR) imaging, on the other hand, shows a high signal intensity within the gallbladder wall on T1-weighted images. This is highly indicative of hemorrhagic infarct and necrosis. Despite the issues presented, which make detection difficult, prompt diagnosis and treatment have been shown to make a significant difference, with mortality reaching as low as 5%. These further highlight that early surgical intervention is the key to patient safety [5].

## 2. Case Presentation

An 80-year-old woman was admitted to Dar Al Amal University Hospital complaining of constant pain in the right upper quadrant of the abdomen for approximately 12 hours in duration. The pain was associated with nausea and vomiting, without jaundice or hyperthermia. The patient's past surgical history revealed a bipolar fixation of the left hip for a fracture. Her observations were all within normal ranges.

**Figure 1:**
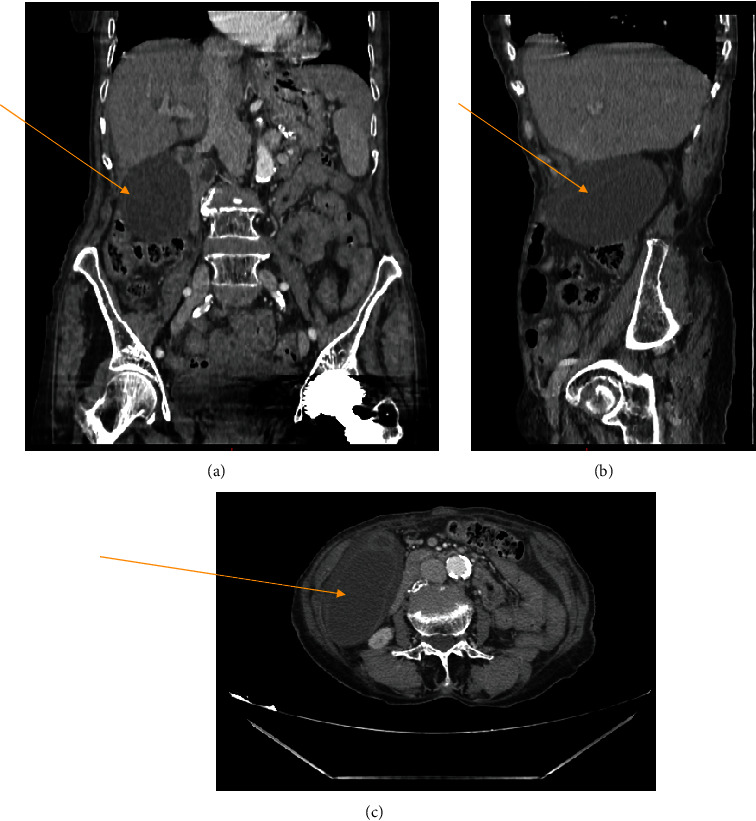
Multislice CT scan of abdominal and pelvic with IV contrast media in 1.25 mm slices thickness.

A focused examination elicited right upper quadrant tenderness and a positive Murphy's sign. Laboratory data showed a high C-reactive protein (CRP) of 336.6 mg/L, but other lab tests such as bilirubin and other liver function tests were within the normal range. It was decided to commence intravenous fluids and broad-spectrum antibiotics. Abdominal ultrasound (US) demonstrated a large cystic lesion in the right infrahepatic region, suggestive of a distended gallbladder with wall thickening without stones. A computed tomography (CT) examination showed the gallbladder to have marked wall edema suggestive of acalculous acute cholecystitis, measuring 10 cm in largest diameter, as shown in Figure 1. Other findings included a CBD which was mildly dilated and measured 10 mm. This also raised the suspicion of a possible cholangitis picture. The liver was also described as being mildly enlarged with a tiny hypodensity measuring 7 mm in size in image C.

**Figure 2:**
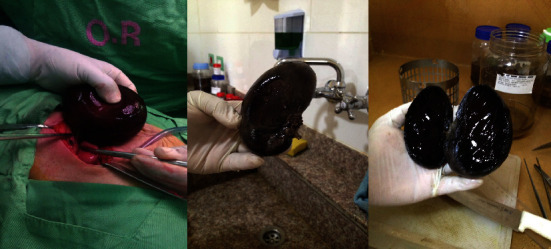
The photos show gross images of the distended and gangrenous gallbladder mass excised during the laparotomy.

The patient was scheduled for laparotomy after presenting with right upper quadrant pain with elevated inflammatory markers and having associated nausea and vomiting. This was decided as compared to laparoscopy to reduce the surgical time and possible deterioration of the patient. Intraoperatively, a distended gangrenous gallbladder, as shown in Figure 2, was seen. A closer examination revealed a counterclockwise torsion of 360 degrees of the gallbladder around its mesentery. The management of this presentation included detorsion and cholecystectomy. Five days after the operation, the patient suffered no complications and was discharged.

## 3. Discussion

The diagnosis of gallbladder volvulus, preoperatively, is difficult. The nonspecific signs and symptoms suggest an acute infective pathology and prompt clinicians to manage it with resuscitation and appropriate antibiotic therapy [6]. Therefore, most cases are diagnosed intraoperatively at present, as was the case here. Laboratory assessments are often nonspecific, as exemplified by our case, whereby only CRP, a nonspecific acute phase reactant, was elevated. Radiological investigations remain nonspecific, with ultrasound imaging often revealing a large, distended floating gallbladder in the infrahepatic region or in a transverse orientation. Additionally, CT findings are also nonspecific, showing a massively distended gallbladder and pericholecystic fluid, as well as other subtle signs such as loss of enhancement and a change in orientation from vertical to horizontal [7]. Recent advances in radiology can confirm the role of coronal magnetic resonance imaging (MRI) and magnetic resonance cholangiopancreatography (MRCP) in the definitive diagnosis of the condition.

It had initially described a triad known as “the triad of triads” which was recommended to aid clinicians in discriminating this condition from acute cholecystitis [8]. It includes inspecting the appearance of the patient for risk factors. These include old age, cachectic appearance, and any deformities of the spine. Secondly, presenting with symptoms of sudden onset right upper quadrant pain which is associated with early emesis is an important indicator to consider. Finally, examination findings such as a palpable abdominal mass, a toxic presentation, and a pulse-temperature discrepancy are also important to look out for.

Particularly in our case, CT imaging suggested a diagnosis of acalculous cholecystitis or cholecystitis without a stone. Acalculous cholecystitis accounts for 10% of all cases of acute cholecystitis [9]. The condition is usually attributed to gallbladder dysfunction secondary to several factors such as long periods of fasting, total parenteral nutrition (TPN), or drastic weight loss. This hypokinetic function after major surgery, for example, results in a concentration of bile salts and a buildup of intraluminal pressure within the organ, which can consequently result in ischemia and necrosis from the pressure and perforation of the organ. This diagnosis can be made using ultrasound imaging or CT imaging showing a significantly thickened wall with edema and possibly pericholecystic fluid, as was the case here. However, an additional diagnostic test which was not employed in this case is the hepatobiliary iminodiacetic acid (HIDA) scan with administering cholecystokinin (CCK). This test is useful when diagnosing chronic acalculous cholecystitis in stable patients when cholecystitis is suspected despite equivocal imaging and allows one to demonstrate a blockage of the cystic channel. In this acute scenario, it was not deemed necessary for management.

The diagnosis of this disease should be considered in all patients (especially elderly women) presenting with right upper quadrant pain (RUQ) that is unremitting despite adequate medical treatment. Our case was diagnosed intraoperatively, but it is recommended that, when suspected, gallbladder volvulus should be diagnosed as soon as possible. This is essential to prompting early surgical exploration. If not, it can result in further unresponsiveness and deterioration of patients. It is also important to consider other possible differentials which can lead to similar presentations of deterioration such as cholecystitis with an unidentified stone or other causes of blockage of the cystic duct which can also develop into a necrotic or perforated gallbladder. Early diagnosis and treatment can eliminate many sequels such as gallbladder rupture and biliary peritonitis.

The strength highlighted in our case includes revealing risk factors that should be considered when suspecting a case of gallbladder volvulus to reinforce the need for early diagnosis and improve the prognosis for patients. Nonetheless, requesting high-resolution imaging for early diagnosis of the suspected case using MRI and MRCP is practically more difficult to achieve in the hospital environment. This would be a preferable imaging modality for stable patients. This was exemplified using CT imaging to provide more nonspecific findings which could potentially delay intervention in such critical cases. Despite a laparotomy being performed in this case, a laparoscopic approach remains easy and feasible and is the first choice for intervention in this case. Given the potential deterioration of the patient and the availability of resources in the Lebanese hospital, a laparotomy was undertaken. Detorsion is not always necessary if structures are well identified.

## 4. Conclusion

Despite the disease being known for centuries, the management has not significantly changed. A gallbladder torsion is a rare surgical emergency which requires a high index of suspicion to diagnose the condition preoperatively and to intervene promptly. Optimal patient outcomes are seen when combining early diagnostic imaging and surgical intervention to remove the gallbladder. Emergency surgery as a treatment option should be carried out based on clinical deterioration and suspicion of complicated cholecystitis. Gallbladder volvulus could be kept in mind but it is not necessary, even futile, useless, and potentially deleterious in terms of further delay.

## Abbreviations

MR: Magnetic resonance
CRP: C-reactive protein
US: Ultrasound
CT: Computed tomography
MRI: Magnetic resonance imaging
MRCP: Magnetic resonance cholangiopancreatography
RUQ: Right upper quadrant.
